# Spatial Myeloid Landscape of Large Artery Atherosclerotic and Cardioembolic Thrombi Retrieved by Mechanical Thrombectomy

**DOI:** 10.1096/fj.202501658RR

**Published:** 2025-12-02

**Authors:** Sugyeong Jo, Taedong Ok, Chae Min Lee, Jae Woong Jeong, Yun Ju Lee, Sung Jae Shin, Sungsoon Fang, Kyung‐Yul Lee, Bo Kyung Yoon

**Affiliations:** ^1^ Graduate School of Medical Science, Brain Korea 21 Project Yonsei University College of Medicine Seoul Republic of Korea; ^2^ Department of Biomedical Sciences, Gangnam Severance Hospital Yonsei University College of Medicine Seoul Republic of Korea; ^3^ Department of Neurology National Health Insurance Service Ilsan Hospital Goyang Republic of Korea; ^4^ Department of Neurology, Gangnam Severance Hospital Yonsei University College of Medicine Seoul Republic of Korea; ^5^ Institute for Immunology and Immunological Diseases Yonsei University College of Medicine Seoul Republic of Korea; ^6^ Department of Biochemistry, College of Life Science & Biotechnology Yonsei University Seoul Republic of Korea; ^7^ Department of Microbiology and Immunology Yonsei University College of Medicine Seoul Republic of Korea; ^8^ Chronic Intractable Disease for Systems Medicine Research Center Yonsei University College of Medicine Seoul Republic of Korea; ^9^ Severance Institute for Vascular and Metabolic Research Yonsei University College of Medicine Seoul Republic of Korea

**Keywords:** etiology, ischemic stroke, spatial transcriptomics, thrombectomy, thrombi

## Abstract

Cardioembolic (CE) and large artery atherosclerosis (LAA) strokes are two main causes of acute ischemic stroke (AIS), carrying a high risk of recurrence. Studies on the composition of CE and LAA thrombi have produced conflicting results, underscoring the need for further investigation into more effective treatment strategies. While myeloid cells such as neutrophils and monocytes are known to promote thrombosis in cardiovascular diseases, their specific roles in thrombosis across AIS subtypes remain unclear. We enrolled 42 AIS patients undergoing endovascular thrombectomy and retrieved their thrombi. Among 42 AIS patients undergoing endovascular thrombectomy, only thrombi and clinical data from patients with large artery atherosclerosis (LAA, *n* = 8) or cardioembolic (CE, *n* = 27) stroke were included in this study, focusing on the two major etiologic subtypes of AIS. Spatial transcriptomic profiling was performed on thrombi from a subset of these patients (LAA, *n* = 4; CE, *n* = 4) to investigate the molecular characteristics of myeloid cells. Immunohistochemistry (IHC) was conducted on thrombi from all included patients (LAA, *n* = 8; CE, *n* = 27) to quantify immune cell populations. Molecular profiling revealed distinct immunological activity between subtypes, despite the absence of statistically significant differences in immune cell abundance by IHC. LAA thrombi exhibited a profibrotic profile, with CD163+ macrophages showing upregulated TGF‐β pathway. scRNA‐seq analysis also revealed an enrichment of profibrotic macrophages in symptomatic atherosclerotic plaques, consistent with spatial transcriptomic findings from atherosclerotic thrombi. In contrast, CE thrombi exhibited increased neutrophil activation and NET formation, with elevated CXCR4 expression in neutrophils. Our findings suggest that the TGF‐β‐mediated profibrotic activity of macrophages and CXCR4‐driven NET formation in neutrophils are associated with distinct patterns of immunothrombosis in LAA and CE strokes, respectively. These observations could contribute to the development of etiology‐specific therapeutic strategies.

## Graphical abstract

This study employed GeoMx digital spatial profiling of thrombi retrieved by mechanical thrombectomy from ischemic stroke patients. Spatial transcriptomic analysis revealed distinct immune signatures, suggesting subtype‐specific mechanisms of immunothrombosis. Neutrophil extracellular trap–related pathways were enriched in cardioembolic thrombi, whereas macrophages in large artery atherosclerotic thrombi upregulated fibrosis‐associated genes.

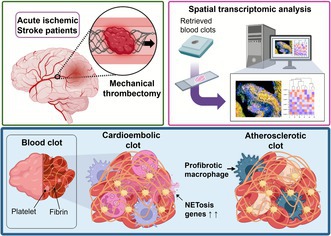

**Abbreviations**
AIS: acute ischemic stroke
CE: cardioembolic
Cit‐H3: citrullinated histone H3
DSP: Digital Spatial Profiling
EVT: endovascular thrombectomy
HbA1c: glycated hemoglobin
ICA: internal carotid artery
IQR: interquartile range
LAA: large‐artery atherosclerosis
MCA: middle cerebral artery
MPO: myeloperoxidase
NE: neutrophil elastase
NET: neutrophil extracellular trap
NIHSS: National Institutes of Health Stroke Scale
ROIs: regions of interest
TOAST: Trial of ORG 10172 in Acute Stroke Treatment

## 1 Introduction

Stroke ranks as one of the leading causes of mortality globally, with ischemic strokes comprising the majority of all new cases and resulting in approximately 63.48 million disability‐adjusted life years and 3.29 million deaths annually [1]. Etiological diagnosis of acute ischemic stroke (AIS) subtypes, primarily using the TOAST (Trial of Org 10172 in Acute Stroke Treatment) classification, is crucial for developing effective strategies to prevent recurrence [2]. Based on this etiologic classification, antithrombotic therapy for secondary prevention is guided by randomized trials and major guidelines. For cardioembolic stroke, direct oral anticoagulants show efficacy and safety advantages relative to warfarin [3, 4]. This evidence aligns with the mechanism of fibrin‐rich thrombi formed in a hypercoagulable state described by Virchow's triad [5]. For non‐cardioembolic stroke, including large‐artery atherosclerosis, antiplatelet therapy is recommended.

In the last decade, endovascular thrombectomy (EVT) in patients with large vessel occlusion strokes has not only significantly improved clinical outcomes but also facilitated the histological, biochemical, and structural analysis of retrieved clots [6, 7, 8, 9, 10]. These analyses have shown that the composition of the thrombus, which includes red blood cells (RBCs), white blood cells (WBCs), fibrin, and platelets, is closely linked with AIS etiology, EVT recanalization rates, stroke severity, and functional outcomes [11, 12]. However, despite extensive research, a considerable number of studies exhibit conflicting outcomes regarding the composition of thrombi and their etiology [13]. As these molecular pathways are influenced by the mechanisms involved in clot formation, these inconsistencies emphasize the need to explore the pathways linked to specific cell types within thrombi.

Immunothrombosis links inflammation and the coagulation cascade, forming intravascular clots as a defense against infection or injury. However, aberrant activation in cardiovascular diseases like myocardial infarction and stroke leads to thrombotic disorders [14]. Platelets are key drivers of immunothrombosis, contributing to thrombosis and activating innate immune cells such as neutrophils and monocytes. At sites of vascular injury, platelets adhere to the extracellular matrix and release agonists like ADP and thromboxane A2, further releasing chemokines such as CXCL4 and CCL5, which recruit neutrophils [15, 16]. Platelet–neutrophil interactions, via P‐selectin and HMGB1, promote the formation of neutrophil extracellular traps (NETs), which serve as scaffolds for thrombus formation and are abundant in AIS thrombi [17, 18, 19]. NETs induce thrombin generation by activating coagulation factor XII. Monocytes also interact with platelets through PSGL‐1 and Mac‐1, forming aggregates that promote the release of pro‐inflammatory cytokines like IL‐1β and IL‐6, further driving NET formation [20, 21].

Despite the significant role of innate immune cells in thrombosis, research on thrombi in AIS has primarily focused on thrombus composition, limited by the difficulty in obtaining sufficient nucleated cells for analysis [22, 23]. The low cellular yield and technical challenges in isolating cells from thrombi with high stiffness have made it hard to perform in‐depth molecular studies. Bulk RNA sequencing, which is alternatively used, lacks the spatial resolution necessary to capture the cellular heterogeneity within thrombi, as it averages gene expression across entire samples. However, recent advancements in spatial transcriptomics technologies, such as GeoMx Digital Spatial Profiling (DSP), enable ROI‐based transcriptomic analysis while preserving spatial context. Although GeoMx DSP does not achieve single‐cell or subcellular resolution, it allows molecular characterization of morphologically or phenotypically defined regions within FFPE sections.

In this study, 42 ischemic stroke patients were enrolled, and retrieved thrombi were used to investigate distinct characteristics and the presence of myeloid cells. GeoMx DSP was employed to explore how immune cells contribute to thrombosis across different AIS etiologies. By analyzing different molecular signatures of immune cells between CE and LAA subtypes, we aim to provide insights into the immune mechanisms underlying thrombus formation in AIS.

## 2 Materials and Methods

### 2.1 Data Acquisition

The single‐cell RNA sequencing (scRNA‐seq) datasets (GSE155512, GSE210152, and GSE253903) were obtained from the Gene Expression Omnibus (GEO) database. GSE155512 includes data from 3 human atherosclerotic carotid arteries, GSE210152 contains 6 human carotid plaques, and GSE253903 comprises 12 human atherosclerotic carotid plaques.

The microarray datasets (GSE124026, GSE146882, and GSE58294) were also downloaded from the GEO database. GSE124026 includes blood samples from 26 ischemic stroke patients, GSE146882 contains blood samples from 10 patients with atherosclerosis‐induced ischemic stroke and 10 healthy controls, and GSE58294 provides blood samples from 69 patients with cardioembolic stroke and 23 healthy controls.

### 2.2 Ethics Statement

This study was approved by the National Health Insurance Service (NHIS) Ilsan Hospital Institutional Review Board (IRB‐2022‐09‐025). All procedures were conducted following the authorized protocol, and written consent was received from all participants. This study was also conducted using UK Biobank (UKB) resource under application number 177007.

### 2.3 Patient Recruitment and Sample Collection

We included patients who underwent EVT from December 2022 through July 2024 at NHIS Ilsan Hospital, from whom a sufficient size of thrombi for analysis was successfully retrieved. The retrieved thrombi were immersed in 4% paraformaldehyde for fixation immediately after EVT and stored until use.

### 2.4 Clinical Parameters, Stroke Etiology, and Sample Selection

Patients underwent regular assessments involving brain magnetic resonance imaging, and either magnetic resonance angiography or computed tomography angiography, heart examinations (such as echocardiography, 24‐h wearable EKG, and cardiac computed tomography), along with routine blood analyses. We collected clinical parameters including demographics (age and sex), underlying vascular risk factors (hypertension, diabetes mellitus, dyslipidemia, and smoking history), prior history of stroke, prior history of cardiac disease (coronary artery disease, atrial fibrillation, and heart failure) and stroke severity as per the National Institutes of Health Stroke Scale (NIHSS). Stroke etiology was classified based on Trial of Org 10172 in Acute Stroke Treatment (TOAST) classification. Eight specimens were selected for spatial transcriptomics analysis: four with LAA etiology and four with CE etiology. The initial selection was based on TOAST classification, followed by the identification of thrombus from patients that most accurately represented each etiology, informed by imaging and clinical data. This was done by an interventional neurologist (T.O.).

### 2.5 Histological and Immunohistochemistry

For histological analysis, retrieved thrombi were fixed in 4% paraformaldehyde, embedded in paraffin, and sectioned at 4 μM. The slides were stained with hematoxylin and eosin (H&E) and Masson's Trichrome. H&E staining was performed using Harris hematoxylin solution (merck HX29014375) and eosin Y (Sigma‐Aldrich, E4382). Masson's Trichrome staining was performed using a Trichrome Stain Kit (Sigma‐Aldrich, HT15).

For immunohistochemistry analysis, immunohistochemical staining was performed using formalin‐fixed, paraffin‐embedded (FFPE) thrombi. The 4 μM thick sections were deparaffinized in xylene and rehydrated through an ethanol gradient. Following deparaffination, antigen retrieval was facilitated by heating for 20 min with FLEX Target Retrieval Solution except for anti‐CD42b and anti‐fibrinogen. The slides were then incubated for 10 min with a 3% hydrogen peroxide solution to inhibit endogenous peroxidase, followed by 2 washes with Tris‐buffered saline. The slides were incubated with primary antibodies at room temperature for 1 h and washed three times, followed by secondary antibody incubation at room temperature for 20 min with EnVision anti‐mouse (Dako, K4001) or EnVision anti‐rabbit (Dako, K4003). The primary antibodies used are listed in Table S1. The detection was performed with 3–3′ diaminobenzidine (DAB) hydrochloride (DAKO, K3468) for 5 min. Counterstaining with hematoxylin for 10 min and mounting followed. Images were acquired with an automated slide scanner (Zeiss, Axioscan 7).

### 2.6 Immunofluorescence Staining

Deparaffinized sections from thrombi were heated at 95°C in a FLEX Target Retrieval Solution (Dako, K8005) for 20 min. After antigen retrieval, 3% H_2_O_2_ solution was used for blocking endogenous peroxidase activity. The sections were incubated for 1 h at room temperature with primary antibodies (Table S1). After washing, sections were incubated for 30 min at room temperature with secondary antibodies: donkey anti‐rabbit AF488 (Invitrogen, A‐21206, AB_2535792) or donkey anti‐rabbit AF546 (Invitrogen, A‐10040, AB_2534016). Stained sections were mounted with Vectashield antifade mounting with DAPI (Vector Laboratories, H‐1200, RRID: AB_2336790). Images were acquired with a laser scanning confocal microscope (Zeiss, LSM780).

### 2.7 Imaging Analysis

Image analysis was performed using QuPath and ImageJ software by researchers who were blinded to the clinical information of the patients. The percentage of each thrombus component was quantified by measuring DAB‐positive areas over total areas. The DAB images were acquired using the color deconvolution plugin and the positive area was calculated by an adjusted threshold according to the staining intensity of the respective antibody. The percentage of fibrosis was quantified from Masson's Trichrome stained images by measuring the blue‐stained area over the total area.

### 2.8 Spatial Transcriptomics Analysis (GeoMx Digital Spatial Profiling)

The spatial transcriptomic profiles of acute ischemic thrombi were analyzed using GeoMx Digital Spatial Profiler (DSP). The 4 μM FFPE tissue sections were prepared and hybridized with the Human Whole Transcriptome Atlas (WTA) probes. The tissue slides were stained with fluorescent‐labeled antibodies targeting an immune cell marker (CD45), macrophage markers (CD68, CD163), and neutrophil markers (ELA2, MPO), followed by nucleic acid staining with SYTO 13 or SYTO 83. Isotype controls were used to confirm the specificity of antibody staining (Figure S4). Details of the antibodies are provided in Table S1. After staining, the slides were scanned using the GeoMx DSP instrument and region of interest (ROIs) were selected based on immunofluorescence signals. For macrophages, fluorescence signal saturation from simultaneous CD68 and CD163 staining interfered with nuclear segmentation. ROIs were therefore defined using a single‐marker channel (CD68 or CD163), with nuclei visualized by SYTO 13. For neutrophils, ROIs were selected using combined NE and MPO channels. Oligonucleotides from selected ROIs were released by ultraviolet light, then indexed using Illumina adapters. Libraries were sequenced at 100 bp paired‐end on Illumina NovaSeq6000.

### 2.9 GeoMx Transcriptomic Data Analysis

Each ROI's data were normalized by Q3 value and log_2_‐transformed normalized counts were used for the analyses. Differentially expressed genes (DEGs) between the CE subtype and the LAA subtype were identified with the following parameters: Benjamini–Hochberg adjusted *p*‐value < 0.05 and fold change ≥ 1.5. Gene set enrichment analysis (GSEA) was performed using the GSEA software (version 4.2.3, Broad Institute). Hallmark and reactome gene sets were obtained from the molecular signatures database MSigDB (version 2024.1.Hs) [24, 25]. The gene set permutation number was set to 1000 for all analyses. Normalized enrichment score (NES) ≥ 1, nominal *p*‐value < 0.05 were considered significant enrichment. NET score was calculated by the gene set variation analysis (GSVA) R package (version 1.52.3) [26]. Previously identified neutrophils and NETosis‐related gene sets were used to assess an enrichment score (Table S2) [27, 28].

### 2.10 UK Biobank

Olink data from the UKB was generated by the UK Biobank Pharma Proteomics Project (UKB‐PPP). Using the 10th revision of the International Statistical Classification of Diseases (ICD‐10) coding system, participants diagnosed with ICD‐10 codes I63.0 or I63.4 were initially selected, and those diagnosed with both codes were subsequently excluded. Only cases with available Olink proteomic data were included. To reduce the influence of extreme values, participants with either the highest or lowest expression of CD163 or TGFB1 were considered outliers and excluded from the analysis based on the interquartile range (IQR) method. Following this exclusion, a total of 10 and 45 participants with I63.0 and I63.4, respectively, were included. Protein concentrations are expressed as log_2_‐transformed normalized protein expression (NPX) values.

### 2.11 ScRNA‐Seq Processing

The scRNA‐seq datasets of human carotid atherosclerotic plaques were downloaded from the GEO database. The raw gene expression matrices were reanalyzed using the R package Seurat v5.2.1 [29]. The doublet cells were removed using the Python package Scrublet [30]. The low‐quality cells were filtered out based on the total unique molecular identifiers (UMIs), the number of detected genes, and the percentage of mitochondrial genes. For GSE210152, cells with at least 500 detected genes, fewer than 20 000 UMIs, and mitochondrial gene content below 5% were retained. For GSE155512 and GSE253903, cells with 300 to 4000 detected genes and mitochondrial gene content below 10% were chosen. In GSE253903, cells with UMI counts exceeding 20 000 were also removed. Integration and dimensional reduction were performed using the IntegrateLayers function with the anchor‐based canonical correlation analysis (CCA) integration. Clustering was conducted using the FindNeighbors function with the first 15 CCA components and FindClusters function with a resolution of 0.5. The RunUMAP function was used for visualization in a two‐dimensional space. The R package scCATCH, SingleR, and known marker genes from published articles were used to annotate cell types. For subclustering of monocytes/macrophages, the RunPCA function was performed, followed by the same clustering procedure as applied to the broad cell types.

### 2.12 Single Cell RNA‐Seq Data Analysis

The CytoTRACE package was employed to predict the relative differentiation state of the monocyte_macrophage cluster [31]. Differentiation scores were visualized using the plotCytoTRACE function with UMAP. Intercellular communication analysis was performed independently for each condition (ASYM and SYM) using the CellChat package [32]. The interactions in less than 10 cells were filtered out and the communication probability based on ligand‐receptor interactions was estimated. The two cellchat objects from each condition were merged using the mergeCellChat function for comparative analysis. The rankNet function and netVisual_aggregate function were used to visualize differences in signaling pathways within the c1 subcluster of the monocyte_macrophage cluster. The Gene Ontology (GO) and gene set enrichment analysis of the C1 subset were performed using the FindAllMarkers function in Seurat, clusterProfiler, and enrichplot.

### 2.13 Bulk Data Processing and Analysis

Microarray datasets of blood from ischemic stroke patients were obtained using the R package GEOquery and Biobase. The ComBat function from the sva package was used to remove batch effects after merging log_2_‐normalized datasets. Principal component analysis (PCA) was performed to confirm batch correction.

### 2.14 Statistical Analysis

Statistical analysis was performed using R statistical software (version 4.4.1, R Core Team 2024) or with SPSS (version 30.0, IBM). The unpaired *t*‐test was used to compare means between groups. For data not fitting a normal distribution or two independent groups, the Wilcoxon rank sum test was utilized. Ratios were compared using Pearson's chi‐squared test. *p*‐value < 0.05 was considered statistically significant.

### 2.15 Role of Funders

The funders had no role in the study design, data collection, data analysis, or composition of this manuscript.

## 3 Results

### 3.1 Retrieval of Thrombi and Baseline Characteristics of AIS Patients

From December 15, 2022 to July 31, 2024, a total of 42 AIS patients who underwent mechanical thrombectomy were recruited, and their thrombi were collected. Thrombi from patients with LAA (*n* = 8) and CE (*n* = 27) were included in the analysis. The baseline characteristics of these patients are presented in Table 1. Hypertension was the most prevalent vascular risk factor in CE patients, while dyslipidemia was most common in LAA patients. Diabetes mellitus and a history of smoking were similar between the groups. Among cardiac comorbidities, atrial fibrillation was more frequent in CE than in LAA (*p* < 0.001).

**TABLE 1:** Clinical characteristics of enrolled patients.

	No. (%)		*p*
	CE (*n* = 27)	LAA (*n* = 8)	
Demographics			
Age (years, mean ± SD)	75.3 ± 10.1	66.9 ± 15.6	0.323
Sex, male	15 (55.6)	4 (50)	0.782
Vascular risk factors			
HTN	18 (67)	3 (38)	0.139
DM	6 (22)	2 (25)	0.869
Dyslipidemia	12 (44)	5 (63)	0.369
Smoke	8 (30)	3 (38)	0.674
Cardiac diseases			
Coronary artery disease	9 (33)	2 (25)	0.656
Valvular heart disease	5 (19)	1 (13)	0.692
Atrial fibrillation	25 (93)	0 (0)	< 0.001
Heart failure	7 (26)	0 (0)	0.107
Medication at admission			
Antiplatelet	9 (33)	2 (25)	0.656
Anticoagulant	5 (19)	0 (0)	0.189
Statin	11 (41)	3 (38)	0.869
Lipid profiles (mg/dL, mean ± SD)			
Total cholesterol	141.2 ± 39	190.6 ± 47.4	0.005
Triglyceride	127.4 ± 187.9	155 ± 75.7	0.016
HDL‐cholesterol	46.3 ± 15.1	39.5 ± 11	0.244
LDL‐cholesterol	77.6 ± 30.3	119.4 ± 38	0.003
HbA1c, %	5.8 ± 0.6	6.2 ± 1.3	0.723
Apolipoprotein A1	119.1 ± 29.8^a^	116.6 ± 24.9	0.832
Apolipoprotein B	67.4 ± 19.9^a^	105.1 ± 27.7	< 0.001
Stroke characteristics			
Initial NIHSS score (median, IQR)	16 (9–20)	9 (6.75–15.25)	0.047
IV tPA	10 (37)	1 (13)	0.189
Site of occlusion			
ICA	11 (41)	4 (50)	0.642
MCA	14 (52)	2 (25)	0.181
BA	2 (7)	1 (13)	0.651
PCA	0 (0)	1 (13)	0.062

Abbreviations: BA, basilar artery; DM, diabetes mellitus; HbA1c, glycated hemoglobin; HTN, hypertension; ICA, internal carotid artery; IQR, interquartile range; IV, intravenous; MCA, middle cerebral artery; NIHSS, National Institutes of Health stroke score; PCA, posterior cerebral artery; tPA, tissue plasminogen activator.

^a^ Apolipoprotein A1 and B were available for 26 patients in the CE group.

At admission, the proportions of patients prescribed antiplatelet agents, anticoagulants, and statins were similar between the groups. Lipid profile analysis revealed significantly higher levels of total cholesterol (*p* = 0.005), triglycerides (*p* = 0.016), LDL‐cholesterol (*p* = 0.003), and apolipoprotein B (*p* < 0.001) in LAA compared with CE patients. The median NIHSS score at admission was significantly higher in CE patients than in LAA patients (*p* = 0.047). Sites of occlusion were predominantly in the ICA and MCA in both groups. Representative imaging findings of CE and LAA stroke are shown in Figure S1.

### 3.2 Absence of Differences in Thrombi Content Across Etiologies

**FIGURE 1:**
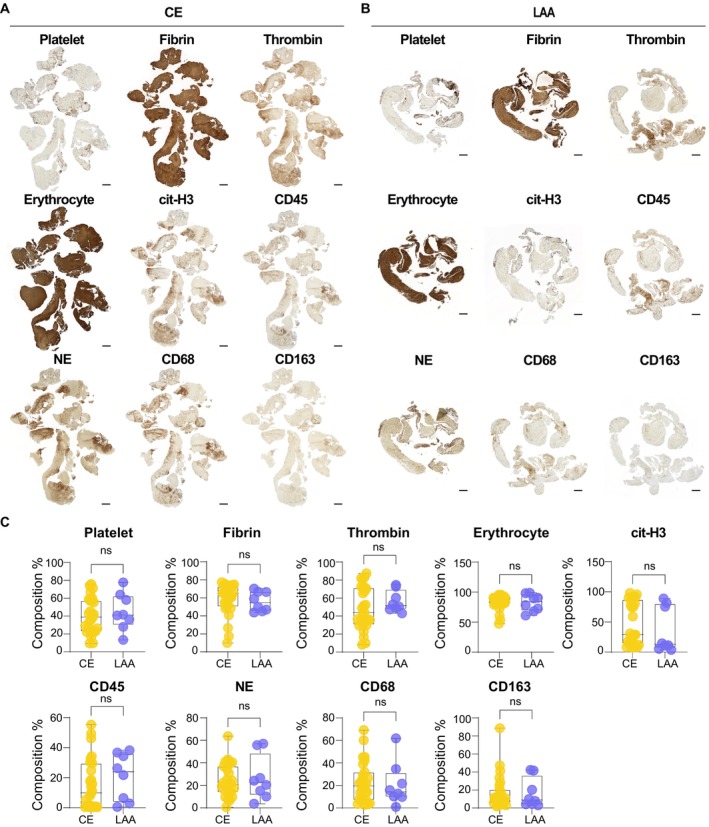
No significant differences in thrombus component expression were observed between CE and LAA subtypes. (A and B) Representative images of IHC staining for platelet, fibrin, thrombin, erythrocyte, cit‐H3, CD45, NE, CD68, and CD163 in CE thrombi (A) and LAA thrombi (B). Scale bars, 1 mm, 250 μm. (C) Quantitative results of IHC staining for platelet, fibrin, thrombin, erythrocyte, cit‐H3, CD45, NE, CD68, and CD163 in CE thrombi (*n* = 27) and LAA thrombi (*n* = 8). Box‐and‐whisker plot shows the median, 25th and 75th percentiles, and whiskers show min to max. Student's *t* test. ns, not significant.

To investigate the thrombus components of the two main AIS subtypes, CE and LAA, immunohistochemical (IHC) staining was conducted. Quantification of the primary components, including platelets, fibrin, and RBCs, showed that fibrin and RBCs constituted a larger proportion of thrombus composition in both subtypes compared to other components (Figure 1A,B). However, there were no significant differences in the proportions of these components between the CE and LAA subtypes (Figure 1C). The median percentage of fibrin was 63.43% (IQR 48.81%–72.34%) in CE and. 54.48% (IQR 45.41%–66.62%) in LAA. For platelets, the median percentage was 39.88% (IQR 22.74%–58.58%) in CE and 41.18% (IQR 29.96%–62.52%) in LAA, with no significant differences found between the two subtypes. To confirm the presence of myeloid cells within thrombi, markers such as CD45, neutrophil elastase (NE) and citrullinated histone 3 (cit‐H3), CD68 and CD163 were also assessed through IHC staining. For neutrophil and NET analysis, NE and cit‐H3 were evaluated. The median percentage of NE was 20.83% (IQR 14.20%–38.88%) in CE and 22.27% (IQR 11.42%–48.51%) in LAA, showing no significant difference between the two subtypes. The median percentage of cit‐H3 was 29.89% (IQR 14.40%–89.45%) in CE and 12.97% (IQR 6.84%–80.15%) in LAA. NETs were more prevalent in CE thrombi than in LAA thrombi, but this difference was not statistically significant, likely due to the small sample size. For macrophages, CD68 and CD163 were assessed, and no significant differences were observed between the two etiologies. The median percentage of CD68 was 20.44% (IQR 7.26%–37.20%) in CE and 14.32% (IQR 9.90%–31.39%) in LAA, while CD163 was 10.33% (IQR 5.52%–21.91%) in CE and 8.95% (IQR 4.20%–36.28%) in LAA. Overall, these results suggest that differences in molecular characteristics, rather than immune cell composition, are more important between etiologies.

### 3.3 Spatial Transcriptomic Profiling of Immune Cell Gene Expression Within CE and LAA Thrombi

**FIGURE 2:**
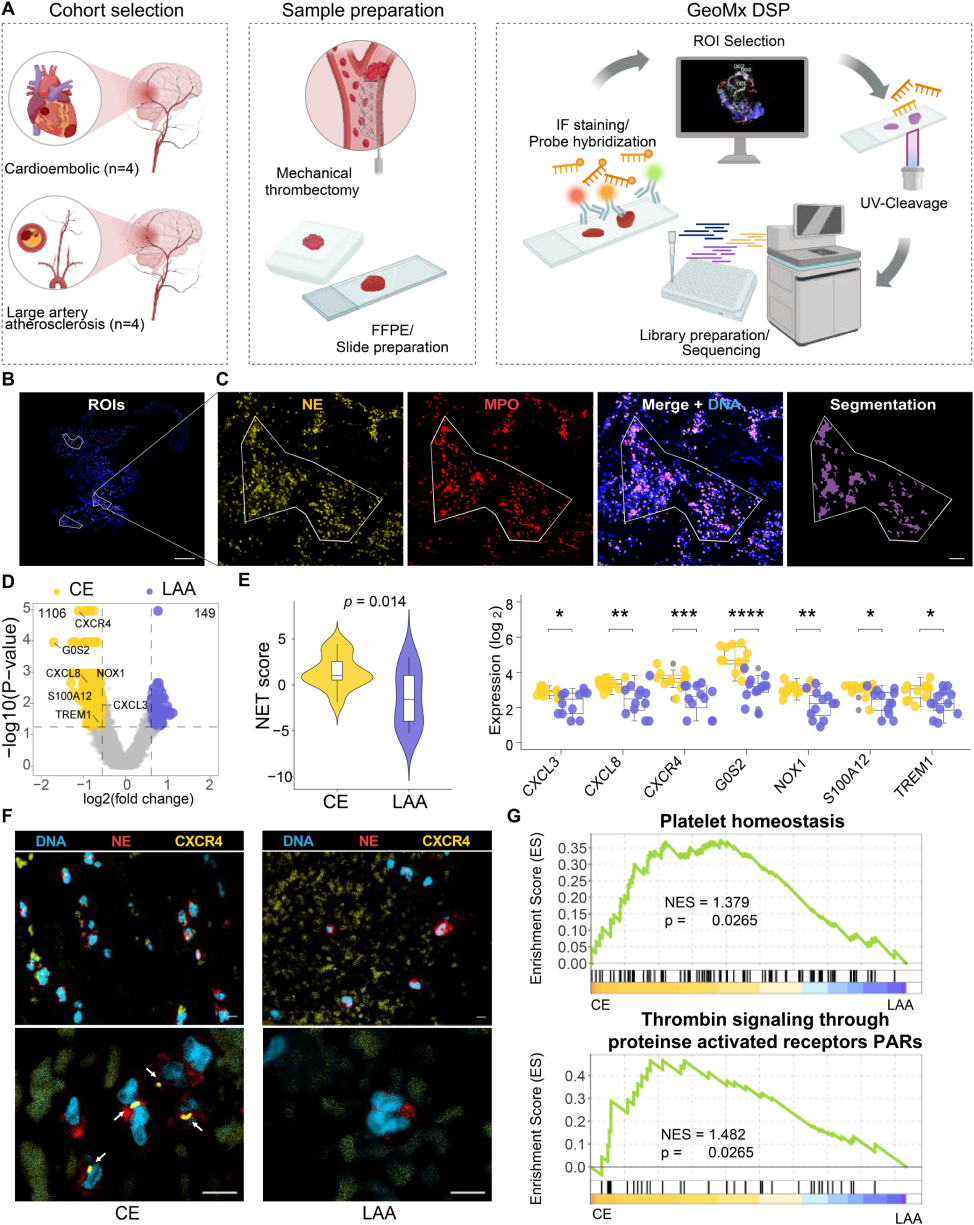
GeoMx spatial profiling reveals neutrophil activation and NET formation in CE thrombi. (A) Schematic representation of the study workflow, including thrombus retrieval, section preparation, and GeoMx Digital Spatial Profiling (DSP). (B) Representative immunofluorescence image of a thrombus showing selected ROIs (white outlines) for neutrophil‐enriched regions. Scale bars, 500 μm. (C) Magnified views of the ROI in panel B, showing NE (yellow), MPO (red), and merged signals with DNA (blue), followed by segmentation masks used to identify neutrophil‐positive areas for quantitative analysis. Scale bars, 100 μm. (D) Volcano plot showing DEGs in NE+ MPO+ regions between CE thrombi and LAA thrombi (*p* < 0.05 and FC > 1.5). (E) Violin plot of NET score on NE+ MPO+ regions. Student's *t* test (left). Box plots showing differential expression of NET‐related genes. Student's *t* test (right). *p < 0.05; **p < 0.01; ***p < 0.001; ****p < 0.0001. (F) Representative images of immunofluorescence staining for DAPI (blue), NE (red), CXCR4 (yellow) in CE thrombi (left) and LAA thrombi (right). White arrows indicate CXCR4 signals within NE^+^ neutrophils. Scale bars, 5 μm. (G) GSEA enrichment plots for “platelet homeostasis” and “thrombin signaling” gene sets enriched in NE+ MPO+ regions of CE thrombi.

To investigate the relationship between the immune cell expression profile in thrombi and AIS etiologies, GeoMx DSP was conducted using FFPE thrombi (Figure 2A). Four thrombi from each CE and LAA subtype were retrieved for analysis. The samples were stained with innate immune cell markers and regions of interest (ROIs) containing more than 100 nuclei were selected to ensure high‐quality transcriptome data.

For specific analysis of activated neutrophils, samples were stained with NE and myeloperoxidase (MPO). A total of 24 ROIs in eight samples were selected. Segmentation was performed in these ROIs, focusing on NE+ MPO+ cells to target the activated neutrophil population (Figure 2B,C). We then performed differential gene expression analysis of NE+ MPO+ segments to compare gene expression between the CE and LAA subtypes. There were 1255 significant differentially expressed genes (DEGs) between CE and LAA. 1106 genes were significantly upregulated in CE samples (Figure 2D).

### 3.4 Increased Activation of Neutrophils in CE Thrombi

Activated neutrophils release NETs, which contribute to thrombus formation promoting coagulation and platelet activation [33]. To assess the difference in neutrophil activation between the two etiologies, we calculated the NET score using previously reported NET‐related genes [27, 28]. The neutrophil NET score was significantly higher in the CE subtype (Figure 2E). Consistent with this result, we observed G0S2, which is expressed in activated mature neutrophils, was most upregulated in the CE subtype [34]. In addition, upregulation of genes involved in neutrophil chemotaxis (*CXCR4*, *CXCL8*, *CXCL3*) and neutrophil activation (*NOX1*, *S100A12*, *TREM1*) was identified in the CE subtype. In particular, CXCR4^hi^ neutrophils drive thrombosis in cardiovascular disease by promoting platelet production [35]. Immunofluorescence analysis confirmed that CXCR4 and G0S2 were overexpressed in the CE subtype compared to LAA (Figure 2F and Figure S3). Furthermore, gene set enrichment analysis (GSEA) of Reactome pathways revealed that platelet homeostasis and thrombin signaling through proteinase activated receptors (PARs) were enriched in the CE subtype (Figure 2G). Overall, these results indicate that neutrophils are more activated in the CE subtype, leading to increased thrombus formation through enhanced NET release and platelet activation.

### 3.5 Profiling of Macrophage Gene Expression Based on Etiology by Spatial Transcriptomics

**FIGURE 3:**
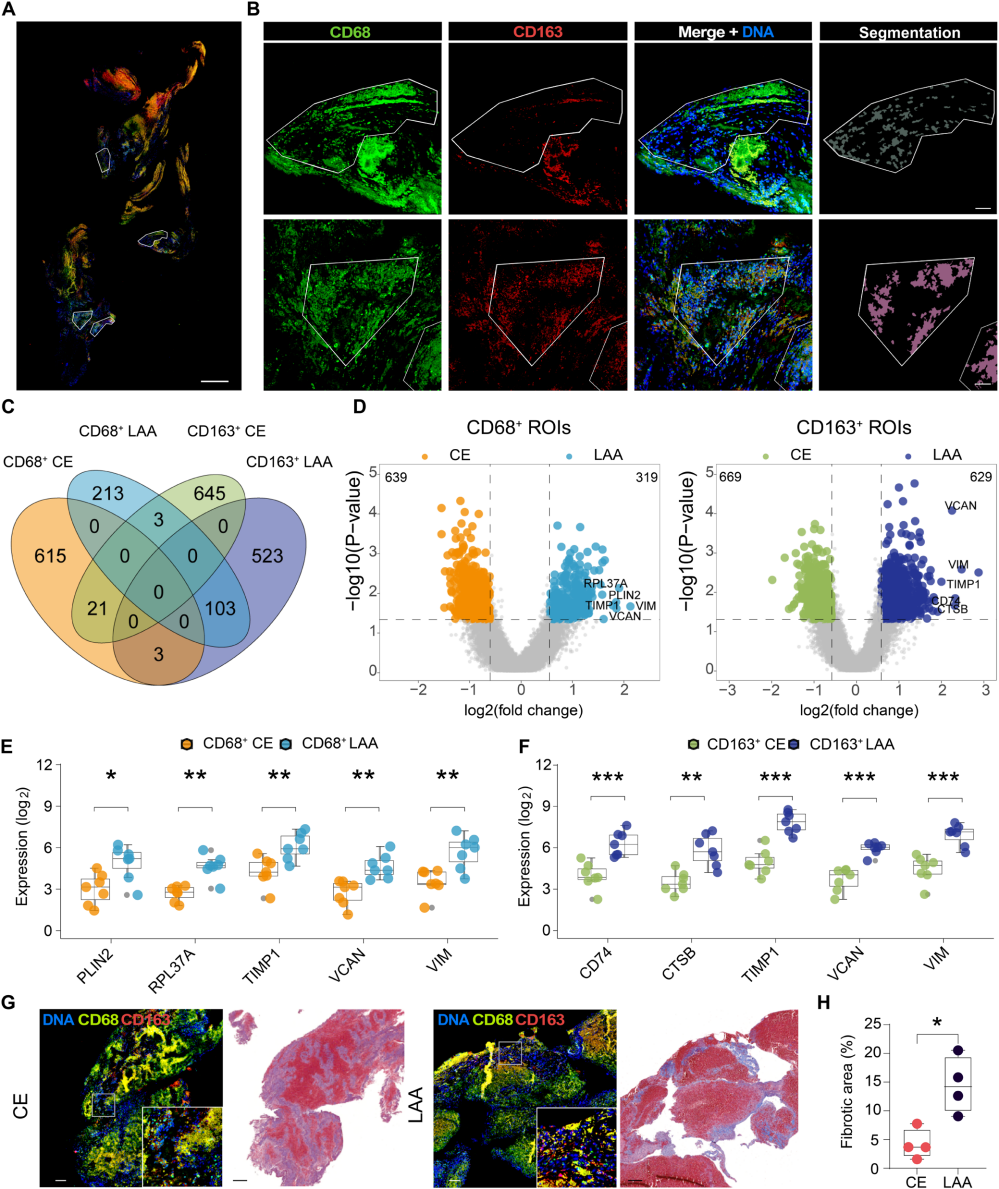
Spatial transcriptomic analysis reveals profibrotic characteristics of macrophages in LAA thrombi compared to CE thrombi. (A) Representative immunofluorescence image of a single thrombus section from one patient, showing CD68 (green) and CD163 (red) staining overlaid with DNA (blue). White outlines indicate selected ROIs enriched for either CD68^+^ or CD163^+^ cells. Scale bars, 500 μm. (B) Representative immunofluorescence images of spatially defined ROIs enriched for CD68^+^ macrophages (upper row) or CD163^+^ macrophages (lower row) in thrombus tissue. Columns show CD68 channel (green), CD163 channel (red), merged image with DNA (blue), and segmentation masks of target cells within the ROI. ROIs were defined using a single‐marker channel to avoid nuclear signal saturation. Scale bars, 50 μm. (C) Venn diagram showing the distribution of differentially expressed genes (DEGs) in CD68+ and CD163+ macrophages across CE and LAA thrombi (*p* < 0.05 and FC > 1.5). (D) Volcano plots showing DEGs in CD68+ (left) and CD163+ (right) macrophages between CE and LAA subtypes (*p* < 0.05 and FC > 1.5). (E and F) Box plots of top‐upregulated genes in CD68+ (E) and CD163+ (F) macrophages of LAA subtypes. Student's *t* test. *p < 0.05; **p < 0.01; ***p < 0.001. (G) Representative images of immunofluorescence staining for DNA (blue), CD68 (green), CD163 (red) and Masson's trichrome staining for fibrosis in CE (left) and LAA (right) thrombi. Gray boxes indicate the selected zoom‐in areas. Scale bars, 200 μm. (H) Box plot showing quantification of the percentage of fibrotic areas in the histological sections. *N* = 4 in each group. Mann–Whitney test. *p < 0.05.

To explore the distinct transcriptomic profiles of macrophages in thrombi from the CE and LAA subtypes, four samples from each subtype were stained with CD45, CD68, and CD163. A total of 28 ROIs were selected based on CD45 expression and further segmented into CD68^+^ (CE, *n* = 7; LAA, *n* = 7) and CD163^+^ (CE, *n* = 7; LAA, *n* = 7) groups (Figure 3A,B). Due to fluorescence signal saturation when using CD68 and CD163 simultaneously, segmentation was performed with a single‐marker channel (CD68 or CD163) in combination with nuclear detection. We then performed differential gene expression analysis by comparing CD68^+^ macrophages between the CE and LAA subtypes, and separately comparing CD163^+^ macrophages between CE and LAA subtypes (Figure 3C,D). A total of 958 significant DEGs (*p* < 0.05, FC > 1.5) were identified in CD68^+^ macrophages, with 319 genes upregulated in the LAA subtype and 639 genes upregulated in the CE subtype. For CD163^+^ macrophages, 1298 significant DEGs were identified, with 629 genes upregulated in the LAA subtype and 669 genes in the CE subtype. For example, lipid droplet‐associated *PLIN2* was significantly upregulated in CD68^+^ macrophages of LAA subtypes, suggesting these macrophages are involved in differentiation into foam cells or exhibit enhanced lipid accumulation (Figure 3E and Figure S5A). In addition, fibrotic marker genes such as *TIMP1*, *VCAN*, and *VIM* were significantly upregulated in both CD68^+^ and CD163^+^ macrophages of the LAA subtype compared to the CE subtype (Figure 3E,F and Figure S5B–D). Consistent with these results, Masson's trichrome staining demonstrated increased fibrosis in LAA thrombi compared to CE thrombi, suggesting that macrophages in LAA thrombi are involved in fibrosis‐related processes (Figure 3G,H).

**FIGURE 4:**
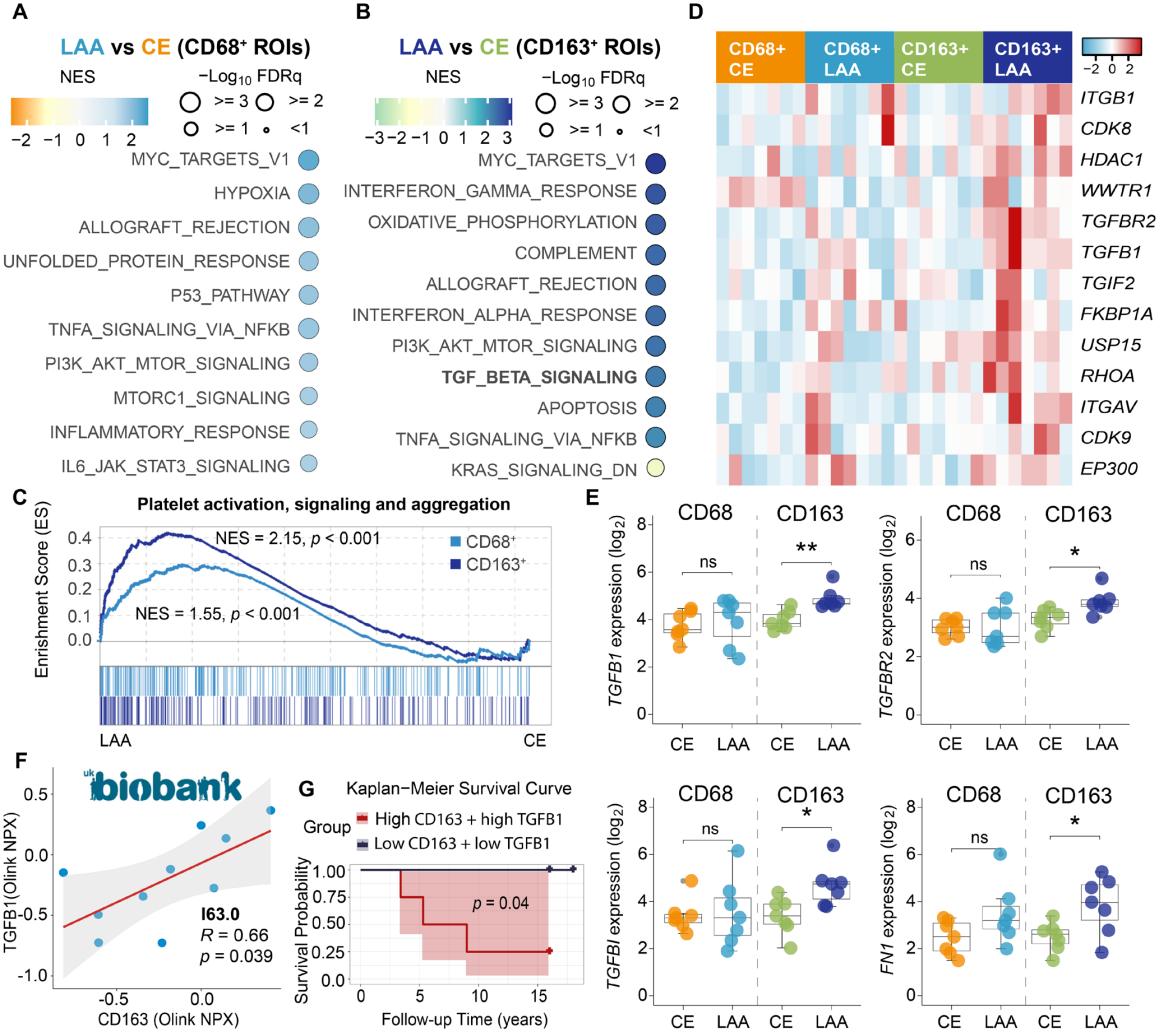
TGF‐β signaling and profibrotic gene expression are enriched in CD163+ macrophages of LAA subtypes. (A and B) Bubble plots of top up‐ and downregulated GSEA of hallmark pathways in CD68+ (A) and CD163+ (B) macrophages in LAA subtypes. (C) GSEA enrichment plots of “platelet activation, signaling and aggregation” pathways in CD68+ and CD163+ macrophages, with higher enrichment observed in LAA thrombi. (D) Heatmap of TGF‐β signaling‐related gene expression in CD68+ and CD163+ macrophages across CE and LAA subtypes. (E) Box plots showing expression levels of TGF‐β signaling‐related genes in CD68+ and CD163+ macrophages across CE and LAA subtypes. Student's *t* test. ns, not significant; *p < 0.05; **p < 0.01. (F) Scatter plot showing the correlation between plasma levels of TGFB1 and CD163 in patients with I63.0 from UKB. (G) The Kaplan–Meier survival curve of high‐ and low‐plasma levels of both TGFB1 and CD163 groups in patients with I63.0 from UKB.

We performed GSEA analysis using both the Hallmark and Reactome gene sets to further explore functional differences of macrophages between the CE and LAA subtypes (Figure 4A,B). Hallmark‐based analysis revealed that pathways including MYC_TARGETS_V1, TNFA_SIGNALING_VIA_NFKB, and PI3K_AKT_MTOR_SIGNALING were associated with both CD68+ and CD163+ macrophages in LAA thrombi. The INFLAMMATORY_RESPONSE pathway was among the top pathways in CD68+ macrophages in LAA thrombi. In contrast, the INTERFERON_GAMMA_RESPONSE, OXIDATIVE_PHOSPHORYLATION, and TGF_BETA_SIGNALING pathways were specifically enriched in CD163+ macrophages in LAA thrombi. Reactome‐based analysis revealed that platelet activation, signaling and aggregation were enriched in both CD68+ and CD163+ macrophages of the LAA subtype, suggesting that distinct macrophage subsets in LAA promote thrombosis by interacting with platelets (Figure 4C).

### 3.6 Profibrotic Characteristics in CD163 ^+^ Macrophage‐Enriched Region

The TGF‐β signaling pathway is closely associated with fibrosis, promoting fibroblast activation and inducing endothelial to mesenchymal transition [36]. Notably, the TGF‐β signaling pathway was identified as one of the pathways in CD163^+^ macrophages of the LAA subtype (Figure 4B). CD163^+^ macrophages of the LAA subtype exhibited significantly higher expression of genes involved in the TGF‐β signaling pathway (*TGFB1*, *TGFBR2*, *TGFBI*) compared to both CD68^+^ macrophages from the CE and LAA subtypes and CD163^+^ macrophages from the CE subtype (Figure 4D,E). Additionally, we observed significantly elevated expression of *FN1*, a gene associated with profibrotic macrophages, in CD163^+^ macrophages of the LAA subtype.

Given that activated CD163^+^ macrophages release soluble CD163 into the circulation [37], we next examined whether plasma levels of CD163 and TGFB1 reflect the profibrotic macrophage signature observed in LAA thrombi. After filtering the plasma proteomic data from UK Biobank (UKB) participants diagnosed with I63.0 (cerebral infarction due to thrombosis) or I63.4 (cerebral infarction due to embolism), we assessed the correlation between plasma CD163 and TGFB1 levels (Figure S2A). Plasma levels of CD163 and TGFB1 showed a strong positive correlation in participants with I63.0. In contrast, no significant correlation was observed in participants with I63.4 (Figure 4F and Figure S2B). Kaplan–Meier analyses revealed that high plasma levels of both CD163 and TGFB1 are associated with poor survival in participants with I63.0, but not in those with I63.4 (Figure 4G and Figure S2C). We further analyzed the three microarray datasets (GSE124026, GSE146882, and GSE58294) (Figure S2D–F). The analysis revealed a weak negative correlation between blood CD163 and TGFB1 expression levels in CE patients. In LAA patients, *CD163* and *TGFB1* exhibited a similar correlation pattern to that observed in UKB participants with I63.0, although the correlation was not statistically significant (Figure S2G). Taken together, these results indicate that a subtype‐specific association between CD163 and TGFB1 in LAA links profibrotic macrophage activation in thrombi to systemic molecular signatures.

### 3.7 Profibrotic Characteristics in Macrophages of Atherosclerotic Plaques

**FIGURE 5:**
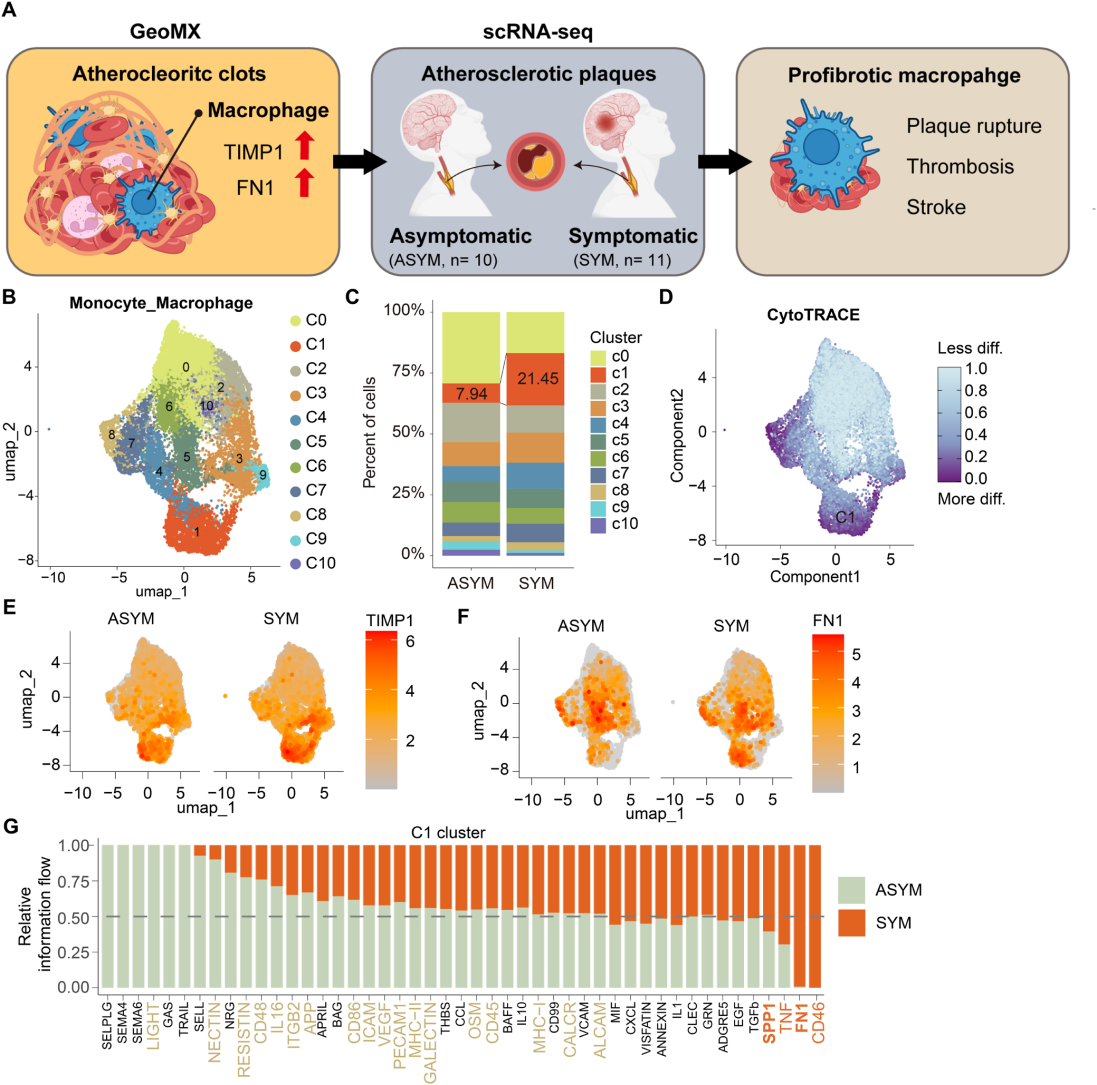
scRNA‐seq analysis reveals an enrichment of profibrotic macrophages in atherosclerotic plaques from symptomatic patients. (A) Schematic illustration of the scRNA‐seq analysis workflow, designed based on spatial transcriptomic findings from atherosclerotic thrombi. (B) UMAP visualization of monocyte/macrophage subsets (*n* = 11) identified by reclustering in atherosclerotic plaques. (C) Stacked bar plot showing the proportion of each cluster across conditions. (D) UMAP showing differentiation potential estimated by CytoTRACE analysis, with lower scores indicating more differentiated states. (E and F) UMAP visualization of fibrosis‐related genes in monocytes/macrophages from asymptomatic (ASYM) and symptomatic (SYM) patients: TIMP1 (E) and FN1 (F). (G) Information flow of outgoing signaling pathways from subset C1 in ASYM and SYM patients. Names of significant pathways unique to each condition are highlighted in color (ASYM, olive; SYM, orange).

Rupture and thrombosis are more frequently observed in carotid atherosclerotic plaques from symptomatic patients, and these are pathologically linked to thrombus formation in LAA stroke [38, 39, 40]. To explore macrophage immune profiles relevant to LAA thrombus formation, we reanalyzed publicly available scRNA‐seq datasets from human atherosclerotic plaques (GSE155512, GSE210152, and GSE253903) (Figure 5A). After quality control with doublet detection, integration, and clustering, each cluster was annotated based on canonical markers (Figure S6A,B). We further reclustered the Monocyte_Macrophage cluster into eleven distinct subsets (Figure 5B and Figure S6C). Subsets C1, C3, C5, C7, and C8 were more abundant in plaques from symptomatic patients compared to those from asymptomatic patients (Figure 5C). Notably, subset C1 was markedly enriched in symptomatic plaques, accounting for 21.5% of the Monocyte_Macrophage population. We also applied CytoTRACE to assess the differentiation status of the myeloid cell population and found that subsets C8, C1, and C7 exhibited the highest differentiation potential (Figure 5D). These highly differentiated subsets, particularly C1, showed elevated expression of fibrosis‐associated genes such as *TIMP1* and *FN1*, consistent with the macrophage phenotype observed in atherosclerotic thrombi (Figure 5E,F). GSEA analysis using GO also revealed that fibrosis‐associated pathways were enriched in subset C1 of symptomatic patients (Figure S6D). To further explore the signaling interactions of this profibrotic population, we performed CellChat analysis focusing on subset C1. Informative flow analysis revealed that fibrosis‐associated pathways, including *SPP1* and *FN1*, were enriched in outgoing signals from subset C1 of symptomatic patients (Figure 5G, and Figure S6E,F). In conclusion, profibrotic macrophages were enriched in both atherosclerotic thrombi and symptomatic atherosclerotic plaques. These findings suggest that profibrotic macrophages may be involved in plaque rupture, thrombosis, and AIS.

## 4 Discussion

Antithrombotic agents have been prescribed based on AIS etiology, with anticoagulants often used for CE stroke and antiplatelet agents for non‐CE stroke such as LAA stroke [2, 41]. However, these strategies remain largely empirical, and the high recurrence rate of AIS is still challenging despite these interventions. Most previous studies on thrombosis mechanisms in AIS subtypes have focused on major component differences, such as fibrin, platelets, and RBCs. Due to the inherent characteristics of thrombi, these studies have been limited to IHC and bulk transcriptomic analyses, often yielding controversial results [13, 42, 43, 44]. Recent advancements in spatial transcriptomics now enable more precise, cell‐specific analyses of the molecular mechanisms involved in thrombosis. In this study, we analyzed thrombi from AIS patients who underwent mechanical thrombectomy to investigate molecular differences and underlying mechanisms between the two major etiologies, CE and LAA. In CE stroke, blood stasis, structural changes in the cardiac chamber, and abnormal changes in blood constituents initiate the coagulation cascade, leading to embolic thrombus formation, while in LAA stroke, inflammation destabilizes plaques, resulting in rupture and atherothrombosis [5, 45, 46, 47]. We identified no significant differences in major thrombus components between the subtypes via IHC analysis of thrombi. Additionally, while innate immune cells involved in immunothrombosis were detected, their proportions did not differ notably. However, immune cell‐specific activity analysis using GeoMx DSP revealed that CE thrombi are characterized by increased neutrophil activation and NET formation, whereas LAA thrombi exhibit a distinct macrophage‐driven profibrotic profile.

NETs are known to promote arterial thrombosis by providing scaffolds that trap platelets and RBCs. Studies on AIS have frequently reported NET markers in blood and cerebral thrombi, suggesting DNase as a potential thrombolytic agent [48, 49, 50]. Genchi et al. and Jabrah et al. reported that CE thrombi contain higher NET levels compared to other AIS subtypes [51]. Although our IHC‐based quantification of cit‐H3 was not statistically significant, the trend of increased NETs in CE thrombi was consistent with these reports, suggesting potential biological relevance. The absence of statistical significance in our analysis is likely related to the limited sample size. This indicates the need for molecular profiling to elucidate neutrophil activation and NET‐related pathways in thrombi. A recent study by Walker et al. [52] demonstrated elevated neutrophil degranulation in CE thrombi using spatial transcriptomic profiling of CD45^+^ leukocytes. Similarly, our analysis identified CE thrombi showing significant upregulation of neutrophil activation including NET formation via spatial transcriptomic profiling of NE^+^MPO^+^ neutrophils. We also observed increased expression of NET‐related genes, such as G0S2, CXCL8, and CXCR4, in CE thrombi. CXCR4^hi^ neutrophils, commonly defined as “aged” neutrophils, are known to exhibit enhanced NET formation and increased aggregation with platelets [53, 54, 55]. Therefore, high CXCR4 expression in neutrophils underscores the importance of aged neutrophils in the pathogenesis of CE stroke. Collectively, our findings of subtype‐specific elevation of CXCR4 highlight the role of neutrophils in promoting thrombus formation and maintaining stability in CE stroke. Furthermore, these findings suggest the potential for neutrophil‐targeted interventions, such as DNase or CXCR4 antagonists like AMD3100, to reduce thrombus formation and improve clinical outcomes specifically in CE stroke.

In addition to neutrophils, monocytes/macrophages are also associated with arterial thrombosis. In atherosclerosis, macrophages contribute to plaque instability and thrombus formation by differentiating into foam cells and forming necrotic cores, which increase the risk of plaque rupture [56, 57]. However, the specific role of macrophages in thrombus formation following plaque rupture remains largely unstudied due to limitations in analyzing rigid thrombi. Recently, Jabrah et al. [58] reported similar levels of CD68^+^ macrophage expression in thrombi from both CE and LAA subtypes. Consistent with this finding, our immunostaining analysis showed no significant differences in the presence of CD68^+^ and CD163^+^ macrophages between CE and LAA thrombi. We referred to CD68^+^ cells as macrophages, in line with the widespread use of CD68 as a pan‐macrophage marker [59, 60]. However, as CD68 is also expressed on monocytes in thrombosis, a contribution from monocytes cannot be excluded.

To further investigate the transcriptomic differences of macrophages across AIS etiologies, we applied spatial transcriptomic profiling targeting CD68^+^ and CD163^+^ macrophages. Profibrotic markers were notably increased in both macrophages in LAA thrombi compared to CE thrombi, suggesting a unique role for profibrotic macrophages in the progression of thrombosis in this etiology. Given the pathological association between LAA thrombi and atherosclerotic lesions [61], we subsequently analyzed single‐cell RNA‐seq data from carotid atherosclerotic plaques. In symptomatic patients, a profibrotic macrophage subset was enriched, consistent with the transcriptional profile observed in LAA thrombi.

Notably, spatial transcriptomic analysis further revealed upregulation of the TGF‐β signaling pathway in CD163^+^ macrophages within LAA thrombi. TGF‐β is a well‐known profibrotic factor that drives tissue fibrosis and endothelial‐mesenchymal transition, promoting atherosclerosis [62, 63]. Furthermore, platelet‐macrophage interactions enhance TGF‐β signaling, contributing to a fibrotic environment [64, 65]. In line with this, we observed enriched pathways for platelet activation, signaling, and aggregation in CD163^+^ macrophages within LAA thrombi. These findings indicate that platelet interactions enhance TGF‐β signaling in this macrophage subset. Recently, Mai H. et al. reported that inhibition of TGF‐β1 significantly delayed thrombus formation in the mouse model [66]. Therefore, the elevated TGF‐β signaling observed in LAA thrombi suggests that CD163^+^ macrophages promote fibrotic remodeling, contributing to thrombosis in this etiology. Collectively, our findings suggest the therapeutic potential of targeting TGF‐β1 signaling to modulate profibrotic macrophage activity and reduce thrombus stability in LAA stroke. Furthermore, given the observed enrichment of platelet activation pathways in CD163^+^ macrophages, combining TGF‐β1 inhibitors with antiplatelet agents produces a synergistic effect to mitigate fibrosis and improve clinical outcomes in LAA stroke.

## 5 Limitations

A limitation of our study is the absence of in vivo animal models to validate the transcriptomic profiling results. The existing thrombosis model, the ferric chloride (FeCl3)‐induced thrombosis model, is not physiologically relevant [67]. In this model, thrombosis rapidly progresses within seconds, limiting its utility for detailed mechanistic investigations. Additionally, there are no established animal models that accurately reflect the specific subtypes of stroke, such as CE and LAA. While atherosclerosis models, including LDL receptor‐deficient (LDLr^−/−^) and apolipoprotein E‐deficient (apoE^−/−^) mice, are commonly used, these models do not reliably progress to thrombus formation [68]. Another limitation is the relatively small number of thrombus samples available for IHC analysis. This limited our ability to detect statistically significant differences in NET marker expression between subtypes, although the trends were consistent with previous reports. Addressing these limitations in future studies will be essential for advancing our understanding of AIS pathophysiology and therapeutic strategies.

## 6 Conclusions

In conclusion, our study highlights distinct immune cell‐specific mechanisms in AIS subtypes. LAA thrombi exhibited a macrophage‐driven profibrotic profile with upregulated TGF‐β signaling, contributing to thrombus stability. In contrast, CE thrombi showed increased neutrophil activation and NET formation, associated with elevated CXCR4 expression. These findings suggest that targeting TGF‐β1 signaling may offer promising therapeutic strategies for LAA strokes, while CXCR4 inhibition could benefit CE strokes, providing etiology‐specific approaches to improve AIS outcomes.

## Author Contributions

S.J., T.O., and B.K.Y. designed the study. S.J., T.O., and B.K.Y. wrote this manuscript and organized the figures. T.O., S.J.S., S.F., and B.K.Y. provided conceptual support and advised on data interpretation. T.O. and K.‐Y.L. contributed to patient recruitment and the collection of clinical samples. S.J., J.W.J., C.M.L., and Y.J.L. performed experiments (image analysis and quantification). C.M.L. analyzed the UK Biobank Resource. S.J. and T.O. analyzed clinical data. S.J. analyzed spatial transcriptomic data, scRNA‐seq data, and microarray data. All authors read the manuscript and approved the submission.

## Funding

This study was supported by grants from the Korea Health Industry Development Institute (KHIDI) (RS‐2024‐00403043), the National Research Foundation of Korea funded by the Korea government (MSIT) (RS‐2024‐00405542), and the Ministry of Health and Welfare (HR18C001202).

## Conflicts of Interest

The authors declare no conflicts of interest.

## Supporting information


**Figure S1:** fsb271283‐sup‐0001‐FigureS1.pdf.

**Figure S2:** fsb271283‐sup‐0002‐FigureS2.pdf.

**Figure S3:** fsb271283‐sup‐0003‐FigureS3.pdf.

**Figure S4:** fsb271283‐sup‐0004‐FigureS4.pdf.

**Figure S5:** fsb271283‐sup‐0005‐FigureS5.pdf.

**Figure S6:** fsb271283‐sup‐0006‐FigureS6.pdf.

**Table S1:** fsb271283‐sup‐0007‐TableS1.docx.

**Table S2:** fsb271283‐sup‐0008‐TableS2.pdf.

## Data Availability

Data that support the findings of this study are available from the corresponding author upon reasonable request. The GeoMx spatial transcriptomics data (raw and third quartile normalized counts) have been deposited in the Gene Expression Omnibus (GEO) database under the accession number GSE283015.
