# Supplementary material for: Spatial Myeloid Landscape of Large Artery Atherosclerotic and Cardioembolic Thrombi Retrieved by Mechanical Thrombectomy
Source: FASEB J. 2025 Dec 2;39(23):e71283. doi: 10.1096/fj.202501658RR (PMC12671477; doi:10.1096/fj.202501658RR)
Supplement: Supplementary file 1 — Figure S1: fsb271283‐sup‐0001‐FigureS1.pdf. [file FSB2-39-e71283-s003.pdf]

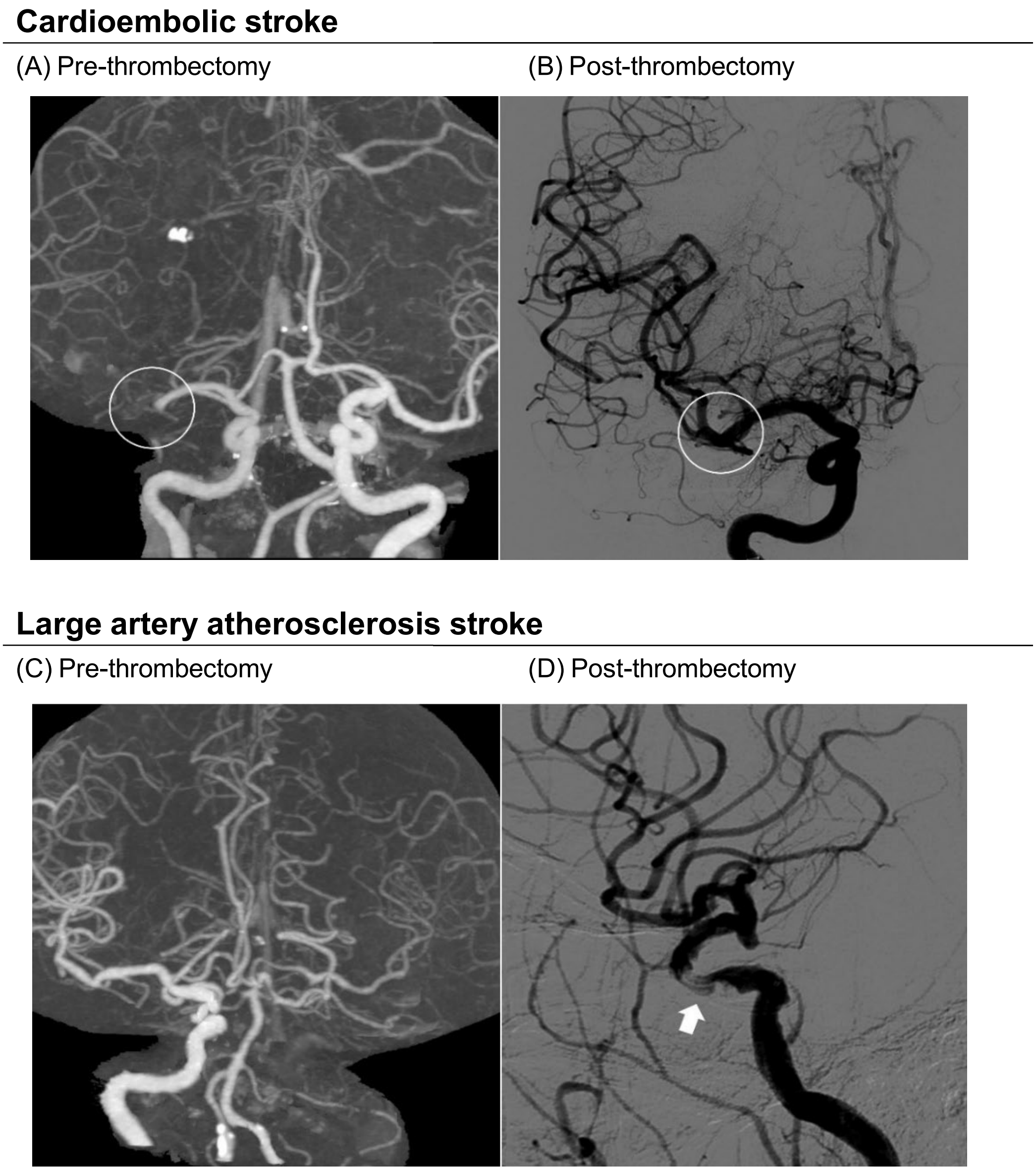

Figure S1. Representative Imaging Findings of Cardioembolic and Large Artery Atherosclerotic Stroke. (A and B) Representative imaging findings of a cardioembolic stroke. (A) Brain computed tomography angiography showing occlusion of the right middle cerebral artery before mechanical thrombectomy. (B) Angiogram demonstrating successful recanalization of the right middle cerebral artery without residual stenosis. The cut-off sign at the right middle cerebral artery (white circle) is indicative of cardioembolism. (C and D) Representative imaging findings of a stroke due to large artery atherosclerosis. (C) Brain computed tomography angiography showing left internal carotid artery occlusion before mechanical thrombectomy. (D) Angiogram illustrating recanalization of the left internal carotid artery with a residual stenotic lesion (white arrow), suggestive of intra-arterial thrombosis secondary to atherosclerosis.
