# Supplementary material for: Spatial Myeloid Landscape of Large Artery Atherosclerotic and Cardioembolic Thrombi Retrieved by Mechanical Thrombectomy
Source: FASEB J. 2025 Dec 2;39(23):e71283. doi: 10.1096/fj.202501658RR (PMC12671477; doi:10.1096/fj.202501658RR)
Supplement: Supplementary file 2 — Figure S2: fsb271283‐sup‐0002‐FigureS2.pdf. [file FSB2-39-e71283-s004.pdf]

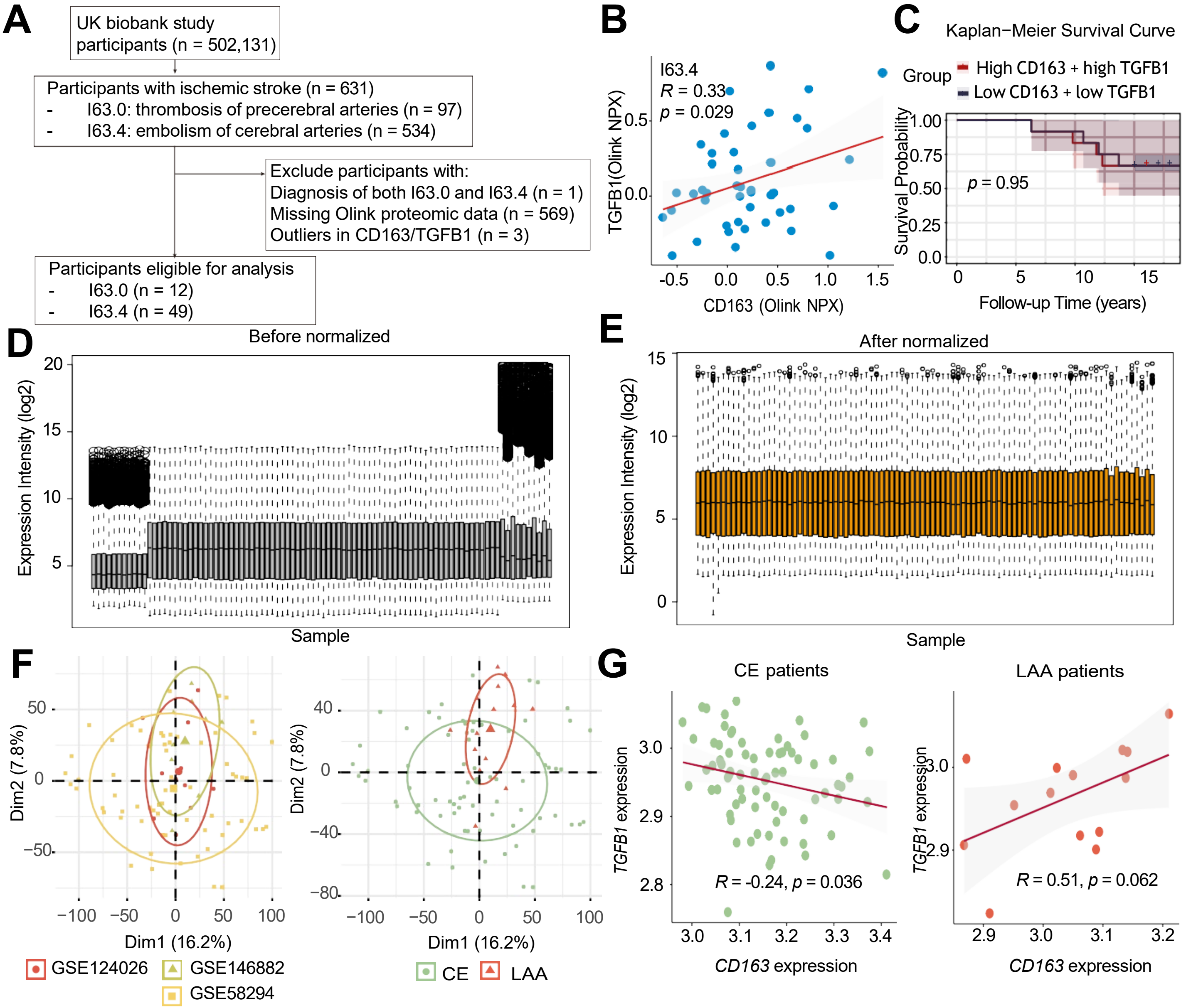

Figure S2. CD163 and TGFB1 levels in plasma and whole-blood transcriptomes of LAA and CE stroke patients. (A) The flow diagram of UKB participant selection. (B) Scatter plot showing the correlation between plasma levels of TGFB1 and CD163 in patients with I63.4 from UKB. (C) The Kaplan-Meier survival curve of high- and low- plasma levels of both TGFB1 and CD163 groups in patients with I63.4 from UKB. (D) Boxplot of gene expression level in whole-blood transcriptome datasets before batch effect removal. (E) Boxplot of gene expression level in whole-blood transcriptome datasets after bath effect correction and normalization. (F) PCA plot of the three datasets after bath effect correction (left), and PCA plot of samples from asymptomatic and symptomatic patients (right). (G) Scatter plot showing the correlation between expression levels of *TGFB1* and *CD163* in CE patients (left) and LAA patients (right).
