# Supplementary material for: Spatial Myeloid Landscape of Large Artery Atherosclerotic and Cardioembolic Thrombi Retrieved by Mechanical Thrombectomy
Source: FASEB J. 2025 Dec 2;39(23):e71283. doi: 10.1096/fj.202501658RR (PMC12671477; doi:10.1096/fj.202501658RR)
Supplement: Supplementary file 3 — Figure S3: fsb271283‐sup‐0003‐FigureS3.pdf. [file FSB2-39-e71283-s006.pdf]

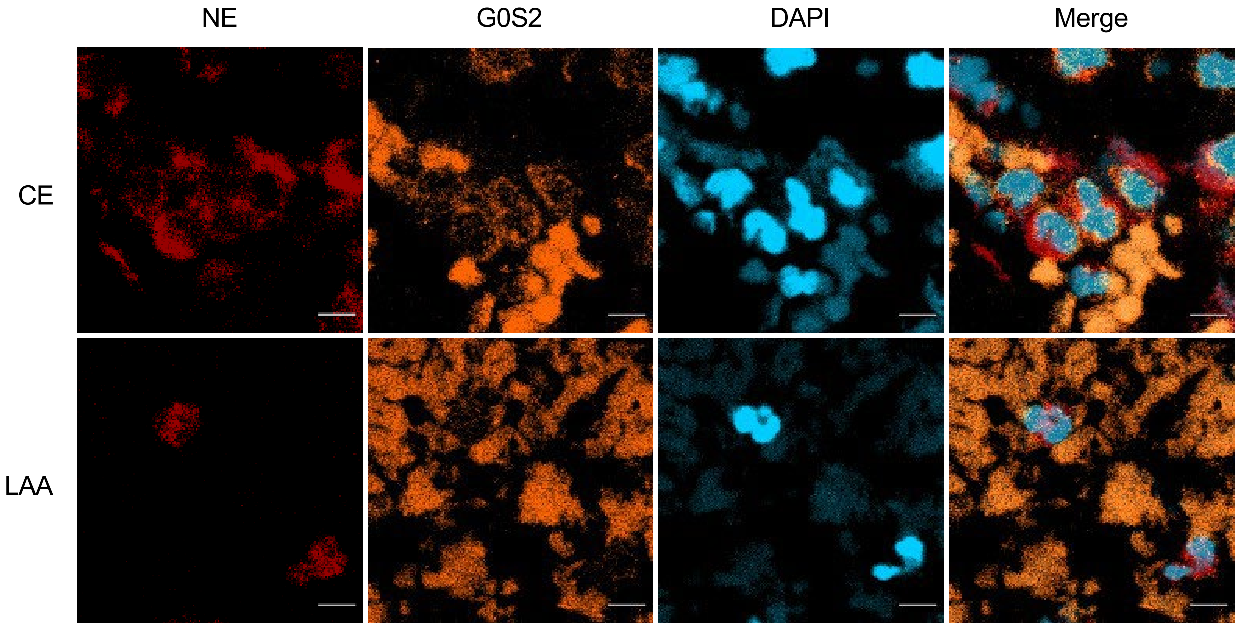

Figure S3. Immunofluorescence staining of G0S2 in neutrophils from CE and LAA thrombi. Representative images of NE (red), G0S2 (orange), and DAPI (cyan) in CE and LAA thrombi, along with merged images. Scale bars, 5 μm.
