# Supplementary material for: Spatial Myeloid Landscape of Large Artery Atherosclerotic and Cardioembolic Thrombi Retrieved by Mechanical Thrombectomy
Source: FASEB J. 2025 Dec 2;39(23):e71283. doi: 10.1096/fj.202501658RR (PMC12671477; doi:10.1096/fj.202501658RR)
Supplement: Supplementary file 4 — Figure S4: fsb271283‐sup‐0004‐FigureS4.pdf. [file FSB2-39-e71283-s001.pdf]

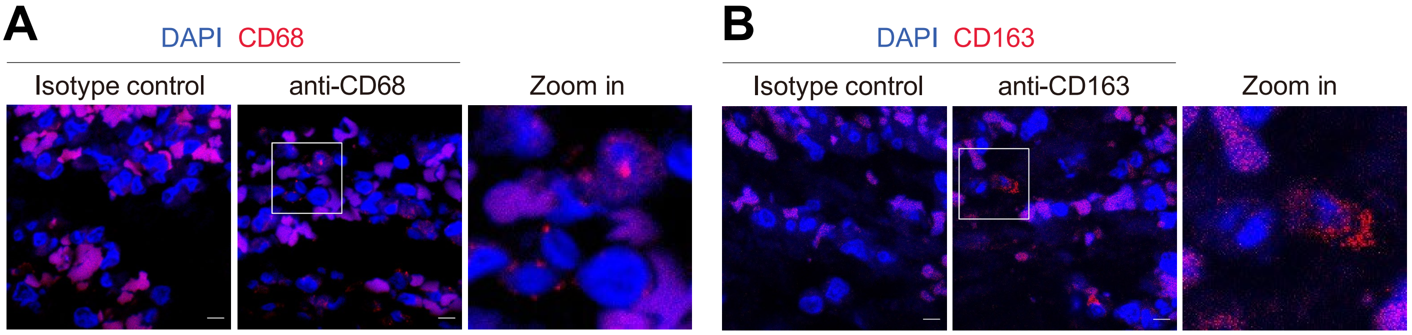

Figure S4. Validation of macrophage markers for spatial transcriptomic analysis. (A) Representative images of immunofluorescence staining for CD68 (red) and nuclei counterstained with DAPI (blue) in thrombus sections. Panels show the isotype control-PE (left), anti-CD68-PE staining (middle), and a magnified view of the region indicated by the white box (right). Scale bars, 5 μm. (B) Representative images of immunofluorescence staining for CD163 (red) and nuclei counterstained with DAPI (blue) in thrombus sections. Panels show the isotype control-AF647 (left), anti-CD163-AF647 staining (middle), and a magnified view of the region indicated by the white box (right). Scale bars, 5 μm.
