# Supplementary material for: Spatial Myeloid Landscape of Large Artery Atherosclerotic and Cardioembolic Thrombi Retrieved by Mechanical Thrombectomy
Source: FASEB J. 2025 Dec 2;39(23):e71283. doi: 10.1096/fj.202501658RR (PMC12671477; doi:10.1096/fj.202501658RR)
Supplement: Supplementary file 5 — Figure S5: fsb271283‐sup‐0005‐FigureS5.pdf. [file FSB2-39-e71283-s008.pdf]

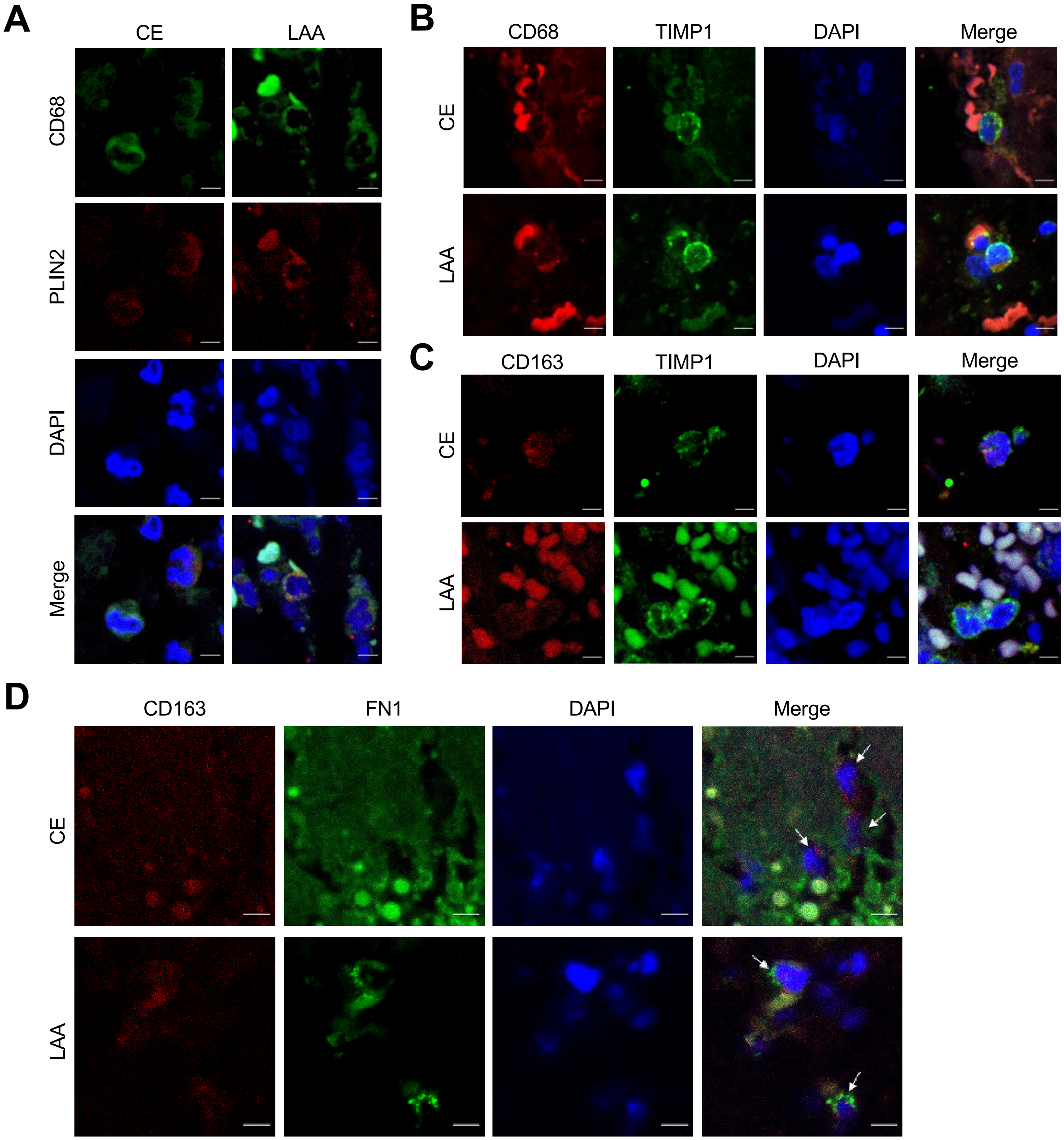

Figure S5. Immunofluorescence staining of indicated macrophage-associated markers in CE and LAA thrombi. (A-D) Representative images showing double staining of CD68 with PLIN2 (A), CD68 with TIMP1 (B), CD163 with TIMP1 (C), and CD163 with FN1 (D). White arrows indicate CD163^{+} macrophages co-expressing FN1. Nuclei were counterstained with DAPI (blue). Scale bars, 5 μm.
