# Supplementary material for: Spatial Myeloid Landscape of Large Artery Atherosclerotic and Cardioembolic Thrombi Retrieved by Mechanical Thrombectomy
Source: FASEB J. 2025 Dec 2;39(23):e71283. doi: 10.1096/fj.202501658RR (PMC12671477; doi:10.1096/fj.202501658RR)
Supplement: Supplementary file 6 — Figure S6: fsb271283‐sup‐0006‐FigureS6.pdf. [file FSB2-39-e71283-s005.pdf]

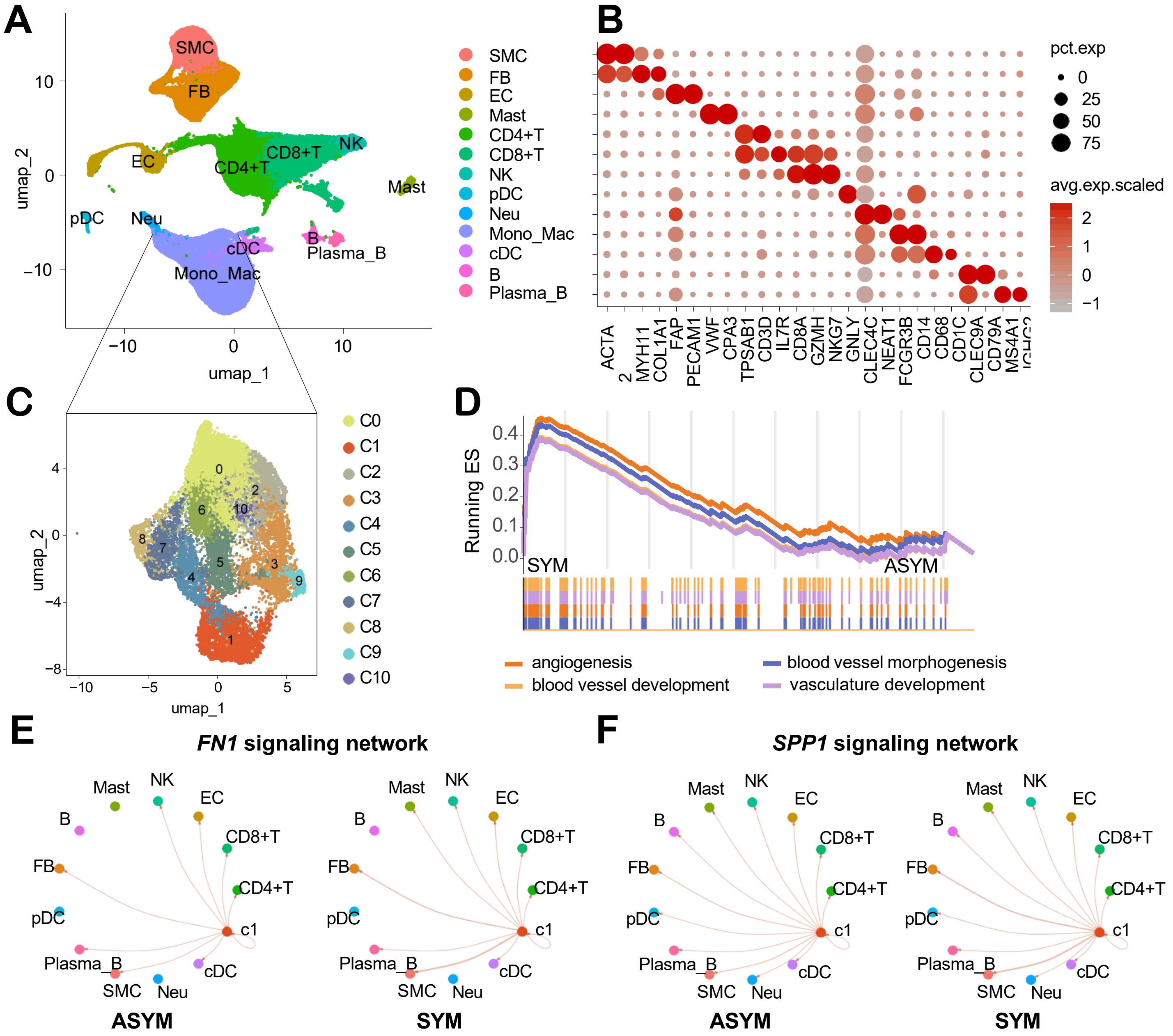

Figure S6. scRNA-seq analysis of carotid atherosclerotic plaques from asymptomatic and symptomatic patients. (A) UMAP visualization of 72,249 cells from carotid plaques. (B) Dot plot for expression of canonical marker genes for each cluster. (C) UMAP plot showing the subclustering of monocyte_macrophage populations into 11 subsets (C0–C10). (D) GSEA enrichment plots of fibrosis-related pathways in subset C1, showing higher enrichment in symptomatic patients. (E and F) Inferred FN1 (E) and SPP1 (F) signaling networks of subset C1 in asymptomatic and symptomatic patients.
