# Supplementary material for: Spatial Myeloid Landscape of Large Artery Atherosclerotic and Cardioembolic Thrombi Retrieved by Mechanical Thrombectomy
Source: FASEB J. 2025 Dec 2;39(23):e71283. doi: 10.1096/fj.202501658RR (PMC12671477; doi:10.1096/fj.202501658RR)
Supplement: Supplementary file 7 — Table S1: fsb271283‐sup‐0007‐TableS1.docx. [file FSB2-39-e71283-s002.docx]

| **Table S1. Antibodies used in the study** | | | | | |
| --- | --- | --- | --- | --- | --- |
| **Target antigen** | **Antibody name** | **Supplier** | **Catalog #** | **RRID** | **Uses** |
| Platelet | Anti-CD42b antibody [EPR6995] | abcam | ab134087 | AB_2936922 | IHC (1:100) |
| Fibrin | Anti-Fibrinogen antibody | abcam | ab34269 | AB_732367 | IHC (1:200) |
| Thrombin | Anti-Thrombin antibody [5G9] | abcam | ab17199 | AB_443712 | IHC (1:400) |
| Erythrocyte | Anti-Glycophorin A antibody [EPR8200] | abcam | ab129024 | AB_2732853 | IHC (1:400) |
| Neutrophil extracellular trap | Anti-Histone H3 (citrulline R2+ R8 + R17) antibody | abcam | ab5103 | AB_304752 | IHC (1:100) |
| CD45 | Purified anti-human CD45 Antibody | Biolegend | 304002 | AB_314390 | IHC (1:100) |
| CD45 | CD45 Antibody (2B11 + PD7/26) [Alexa Fluor® 594] | Novus | NBP2-34528AF594 | AB_3287665 | GeoMx DSP (1:40) |
| Neutrophil | Neutrophil Elastase/ELA2 Antibody (950334) [Alexa Fluor® 594] | Novus | MAB9167AF594 | AB_3148269 | IHC (1:5000)  GeoMx DSP (1:40)  IF (1:200) |
| Neutrophil | Myeloperoxidase antibody [EPR17996] | abcam | ab252131 | AB_2943171 | GeoMx DSP (1:40) |
| CD68 | CD68 (Concentrate) | Dako | M0814 | AB_2314148 | IHC (1:400) |
| CD68 | CD68 (KP1) PE | Santa Cruz | sc-20060 PE |  | GeoMx DSP (1:40)  IF (1:100) |
| CD68 | Alexa Fluor® 488 Anti-human CD68 antibody | Biolegend | 333812 | AB_2074832 | IF (1:50) |
| CD163 | Alexa Fluor® 647 Anti- CD163 antibody [EPR14643-36] - C-terminal | abcam | ab218294 | AB_2943126 | IHC (1:100)  GeoMx DSP (1:40)  IF (1:50) |
| CD163 | Alexa Fluor® 647 anti-human CD163 Recombinant Antibody | Biolegend | 364306 | AB_2922575 | IF (1:50) |
| Mouse IgG1 kappa | Mouse (G3A1) mAb IgG1 Isotype Control (PE Conjugate) | Cell Signaling Technology | 6899 |  | IF (1:100) |
| Rabbit IgG | Rabbit IgG Isotype Control (Alexa Fluor® 647 Conjugate) | Cell Signaling Technology | 3452 | AB_10695811 | IF (1:50) |
| CXCR4 | Anti-CXCR4 antibody [EPUMBR3] | abcam | ab181020 | AB_2910168 | IF (1:200) |
| G0S2 | G0S2 (N-13) | Santa Cruz | sc-133424 | AB_2107211 | IF (1:100) |
| PLIN2 | Perilipin-2/ADFP Antibody [Alexa Fluor® 647] | Novus | NB110-40877AF647 | AB_3176882 | IF (1:100) |
| TIMP1 | Anti-TIMP1 antibody [EPR18352] | abcam | ab211926 | AB_3095674 | IF (1:50) |
| FN1 | Anti-Fibronectin antibody (AB2413) | abcam | ab2413 | AB_2262874 | IF (1:100) |
| IHC: immunohistochemistry; GeoMx DSP: GeoMx Digital Spatial Profiling;  IF: Immunofluorescence**.** | | | | | |
