# Supplementary material for: Spatial Myeloid Landscape of Large Artery Atherosclerotic and Cardioembolic Thrombi Retrieved by Mechanical Thrombectomy
Source: FASEB J. 2025 Dec 2;39(23):e71283. doi: 10.1096/fj.202501658RR (PMC12671477; doi:10.1096/fj.202501658RR)
Supplement: Supplementary file 8 — Table S2: fsb271283‐sup‐0008‐TableS2.pdf. [file FSB2-39-e71283-s007.pdf]

Table S2: NET-related gene list

ACTB	CXCL2	IRF1	PINK1
AGER	CXCR4	ITGAM	PKM
AKT1	CXCR7	ITGB2	PPARGC1A
AKT2	CYBB	KCNN3	PROCR
Ang II	DDIT4	KDM6A	PRTN3
ANXA1	DEFA3	KLF2	PTAFR
ANXA2	DNASE1	LACTOFERRIN	PTGER2
ARPIN	DRP-1	LAMP-2	RIPK1
ATG7	ELANE	LDLR	RIPK3
AZU1	ENO1	LPAR3	Ros1
C1QA	ENTPD4	LYZ	S100A12
C3	EPCAM	MAPK1	S100A8
C3AR1	ERK5	MAPK14	S100A9
C5AR1	F2RL2	MAPK3	S1PR2
CAMP	F3	MCOLN3	SELP
CARD11	FCGR2B	MFN-2	SELPLG
CASP1	FGL2	MIR223	SGK1
CAT	FN1	MIR503HG	SIGLEC14
CCDC25	GSDMD	MMP9	SPP1
CCL2	H2AX	MNDA	SRC
CCL3	H3C14	MPO	STAT3
CCL4	HIF1A	mtDNA	SUCNR1
CCL5	HMGB1	MTOR	SYK
CD177	HRG	MYD88	THBD
CD40L	HSP90	NADPH	TIMP1
CD44	HSPE1	NFE2L2	TKT
CDH1	IL17A	NFIL3	TLR2
CFB	IL1B	NFKB1	TLR4
CFP	IL1RL1	NLRP3	TLR7
CFTR	IL-21	NOX4	TLR8
CLEC4E	IL33	ORAI1	TLR9
CLEC6A	IL36RN	P2RX1	TNF
CLEC7A	IL5	PADI4	TNFAIP3
CSF3	IL6	PARVB	VIM
CTSC	IL8	PF4	WASL
CTSG	ILK	PIK3CA	ZEB1
CXCL1	IRAK4	PIK3CG	
